# Population Genomics Reveals Small‐Scale Metapopulation Structure of Two Strictly Aquatic Keystone Species in a Recently Restored Urban River System (Emscher, Germany)

**DOI:** 10.1002/ece3.71214

**Published:** 2025-04-24

**Authors:** Martina Weiss, Marie V. Brasseur, Armin W. Lorenz, Florian Leese

**Affiliations:** ^1^ Aquatic Ecosystem Research University of Duisburg‐Essen Essen Germany; ^2^ Centre for Water and Environmental Research (ZWU), University of Duisburg‐Essen Essen Germany; ^3^ Aquatic Ecology University of Duisburg‐Essen Essen Germany

**Keywords:** *Gammarus fossarum*, *Gammarus pulex*, intraspecific genetic diversity, mito‐nuclear discordance, population genetics, recolonization, stream restoration

## Abstract

Urbanization and the resulting modifications of freshwater ecosystems can play an important role in shaping metapopulation structure and dynamics of aquatic organisms. Ecological restoration aims at improving river ecosystems by reducing or removing anthropogenic stressors and habitat fragmentation, facilitating natural dispersal among population patches. However, the success of such ecological restoration measures is not guaranteed, and for many of the functionally important but smaller organisms, improved connectivity is difficult to assess. Here, genetic markers can help in assessing small‐scale connectivity and in identifying persisting gene flow barriers. In this study used high‐resolution genetic markers to study the metapopulation structure of two ecologically important amphipod species, *Gammarus pulex* and *Gammarus fossarum*, in the heavily urbanized Emscher catchment in Germany. This catchment was strongly degraded and polluted for over a century but has been restored over the past two decades. For both strictly aquatic species, we analyzed mitochondrial cytochrome c oxidase I (COI) gene sequences as well as nuclear genome‐wide single nucleotide polymorphism (SNP) data. We detected strong metapopulation structure within both species, which was mainly driven by catchment affiliation, wastewater, large in‐stream barriers, and recent recolonization of restored stream sections. However, population structure was not fully explained by these factors, indicating that eco‐evolutionary factors such as priority effects, adaptation, or biotic interactions play a role in shaping the population structure. Furthermore, our data show a strong mito‐nuclear discordance for both species with regard to detailed population structure and also the presence of possible cryptic species for *G. pulex* . Here, nuclear data indicate that the diverging mitochondrial lineages of *G. pulex* (Gp‐C and Gp‐E) represent only one species in this region. Our study shows how genetic markers can support the assessment of population connectivity and thus evaluate the success of ecological restoration.

## Graphical abstract

The study used high‐resolution genetic markers to analyze the metapopulation structure of two aquatic amphipod species, *Gammarus pulex* and *Gammarus fossarum,* in an urban river catchment (Emscher, Germany). Strong metapopulation structure was identified, primarily influenced by catchment affiliation, wastewater, in‐stream barriers, and recolonization, with eco‐evolutionary factors also playing a role. Mito‐nuclear discordance was observed, with nuclear data suggesting that the strongly diverging mitochondrial lineages of *G. pulex* represent a single species in the region. The study demonstrates the power of genetic tools in evaluating restoration success and connectivity.

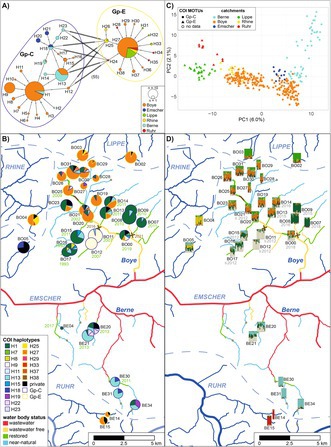

## 1 Introduction

Habitat fragmentation caused by human activities leads to the interruption of gene flow between populations and will consequently modify population structure and genetic diversity (Horreo et al. 2011). This can be explained by the elevated effects of genetic drift and inbreeding in small, isolated populations, resulting in increased population differentiation and reduced genetic diversity (e.g., Bijlsma and Loeschcke 2012; Vrijenhoek 1998). The loss of genetic diversity will impede the ability of populations to adapt and increase their sensitivity to stressor impacts, ultimately threatening the survival of keystone species and heightening the risk of losing associated ecosystem functions (Bijlsma and Loeschcke 2012; Frankham 2005; Hughes et al. 2008; Švara et al. 2022). To preserve genetic diversity, it is therefore important to enhance connectivity and gene flow among populations, especially in urban streams. Here, human‐induced alterations in hydrology, morphology, and water chemistry can significantly impact the metapopulation structure of aquatic organisms (e.g., Miles et al. 2019; Paul and Meyer 2001; Walsh et al. 2005). The increasing awareness of the significance of aquatic biodiversity has resulted in numerous river restoration initiatives including those at heavily modified urban streams (Jähnig et al. 2011), aiming to enhance connectivity among previously isolated source populations and to facilitate recolonization of restored stream sections.

Gene flow between populations depends on several abiotic and biotic factors, such as in‐stream barriers, anthropogenic land use, intra‐ and interspecific competition, and adaptation as well as life history and dispersal traits of the respective species (e.g., De Meester et al. 2002; Fraser et al. 2014). Furthermore, while migration of specimens is required for gene flow, not all migration events lead to genetic exchange, because not all dispersers will survive and reproduce successfully (Bohonak and Jenkins 2003). Therefore, it is not sufficient to purely observe migration to infer genetically effective connectivity. Instead, the population genetic structure must be analyzed to estimate gene flow directly. For this, different genetic marker systems have been established, such as the barcoding fragment of the mitochondrial cytochrome c oxidase subunit I (COI) gene, which is an often‐used, straightforward genetic marker to study intraspecific variation in animals (e.g., Grethlein et al. 2022; Schröder et al. 2022; Weigand et al. 2020). However, this marker aims primarily at verifying morphological species identification and detecting potential cryptic species. To study contemporary gene flow patterns among populations at small geographic scales, higher resolution nuclear markers are needed (Bilton et al. 2001; Freeland et al. 2000). Here, one powerful and cost‐effective method to analyze hundreds to thousands of single nucleotide polymorphisms (SNPs) distributed across the whole genome is double digest restriction site‐associated DNA sequencing (ddRAD‐seq, Peterson et al. 2012; Patterson et al. 2006).

Thus, this method is a candidate target marker system to study metapopulation structure in relatively recently restored urban ecosystems such as the Emscher catchment in the “Ruhr Metropolitan Area” (Western Germany). The Emscher has several larger tributaries such as the Boye and Berne catchments, located north and south of the Emscher, respectively. Both systems differ in the level of restoration, rendering them as interesting study cases to assess and compare connectivity of populations of aquatic species following restoration measurements/actions. While most of the Boye catchment is already restored, parts of the Berne catchment still contain wastewater, and both catchments are separated by the main stem of the Emscher. In the Emscher catchment, different detritivorous key species such as the isopod species *Asellus aquaticus* and *Proasellus coxalis* and the amphipod species *Gammarus pulex* and *Gammarus fossarum* exist. These key species play important roles in organic matter decomposition and their return to previously uninhabitable stream sections is important to establish essential ecosystem functions after restoration. While the two isopod species are more pollution tolerant and might even have survived in the polluted wastewater prior to restoration, the two amphipod species had to immigrate from nearby upstream sections in the river system after restoration, which took up to 8 years (Gillmann et al. 2023). Both amphipod species are sensitive toward organic pollution (e.g., MacNeil et al. 2002; Maltby 1995; Whitehurst 1991) and could therefore not survive in polluted parts of the river system. Despite the high pollution tolerance and quick recolonization of the two isopod species, we detected strong small‐scale population structure within both species and identified persisting migration barriers in the Emscher catchment using high‐resolution genetic markers (Weiss et al. 2024).

In the present study, we aimed to identify dispersal barriers and recolonization pathways after restoration in the urban river system of the Emscher catchment for the two amphipod species *G. pulex* and *G*. *fossarum*. Population genetic studies of both species in other regions have revealed that gene flow within streams is normally high and that smaller in‐stream barriers can be passed frequently enough to maintain gene flow, whereas larger barriers can impede gene flow, and populations of different streams can be highly differentiated (Altermatt et al. 2016; Inostroza et al. 2016; Schneeweiss et al. 2023; Schröder et al. 2022; Švara et al. 2022, 2021, 2019; Weiss et al. 2022; Weiss and Leese 2016). Among both species, *G. pulex* is considered to be less sensitive to a variety of environmental conditions, including, for example, a higher tolerance to lower pH values or lower oxygen (e.g., Adam et al. 2010; Alonso et al. 2010; Peeters and Gardeniers 1998). For both species, several cryptic species are known (e.g., Copilaş‐Ciocianu and Petrusek 2015; Grabner et al. 2015; Lagrue et al. 2014; Wattier et al. 2020; Weiss et al. 2014), which can differ, for example, in their ecological demands or robustness against stressors (Eisenring et al. 2016; Feckler et al. 2014). In the Emscher catchment, only one species of the *G*. *fossarum* species complex (i.e., Type B after Müller 2000; or ABGD‐MOTU 2 after Wattier et al. 2020), but two potential cryptic species of the *G. pulex* species complex, that is, Gp‐C (after Lagrue et al. 2014) and Gp‐E (after Grabner et al. 2015), have been found, which can be distinguished using COI sequences (Grabner et al. 2015). To identify potential cryptic species and to get an overview of the population structure, we first generated COI sequences. We then analyzed high‐resolution genomic SNP data generated with ddRAD‐seq to test if divergent COI lineages represent different cryptic species and to analyze connectivity and gene flow between populations. The chosen sampling sites were the same as for the two isopod species *A. aquaticus* and *P. coxalis* , analyzed in a previous study (Weiss et al. 2024), enabling the comparison of the population structure and detected migration barriers of four hololimnic species with different sensitivities to environmental stressors.

With this, we aimed to test the following hypotheses:

1. Populations of *G. pulex* from different (sub‐)catchments are isolated from each other and exhibit higher genetic diversity within the Boye than within the Berne catchment due to the more advanced ecosystem restoration and a larger number of populations in the Boye catchment. The metapopulation structure will be shaped mainly by wastewater, in‐stream barriers (such as water pumping stations and large weirs), and the recent recolonization of restored stream sections.
2. The population structure of *G*. *fossarum* resembles that of *G. pulex* with isolation of different (sub‐)catchments and isolation caused by wastewater and in‐stream barriers within catchments. Nevertheless, *G*. *fossarum* will exhibit a stronger metapopulation structure and lower genetic diversity due to its greater sensitivity to environmental stressors. Genetic diversity of *G*. *fossarum* populations will be similar between the Boye and Berne catchments because *G*. *fossarum* is more abundant in the Berne, yet this catchment is anthropogenically more strongly impacted and thus more prone to genetic drift effects.

## 2 Materials and Methods

### 2.1 Sampling Area and Sampling Scheme

**FIGURE 1:**
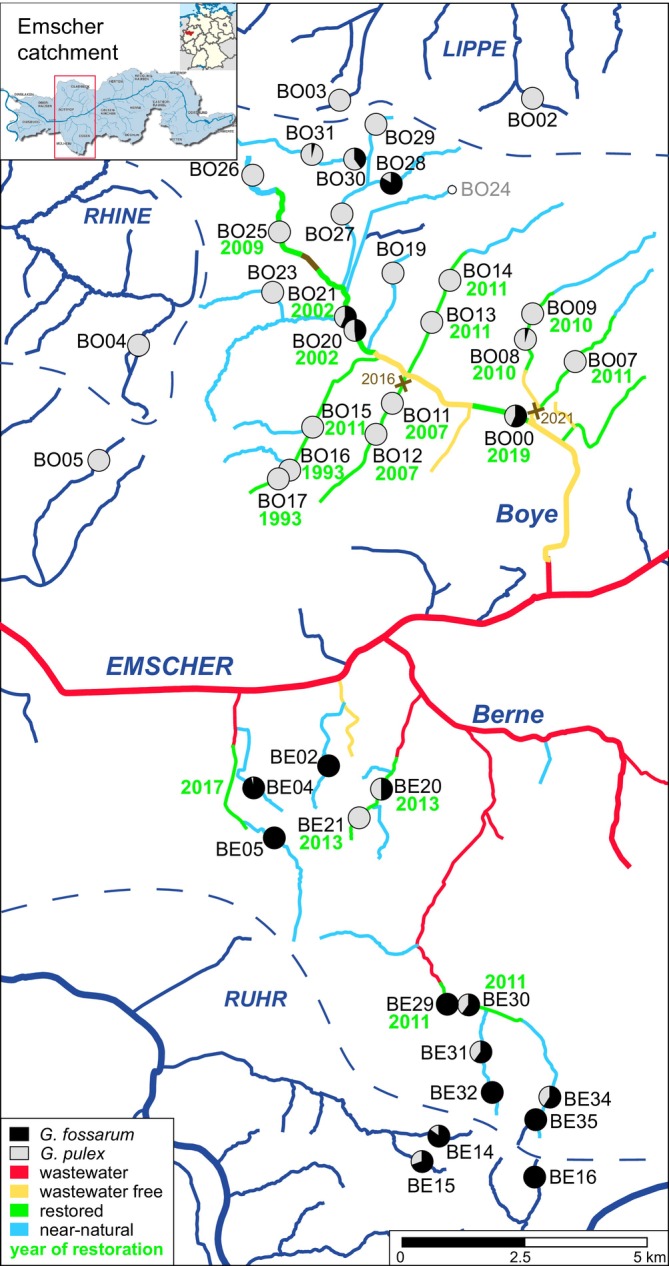
Location of sampling sites with indication of the proportions of *G. pulex* (grey) and *G. fossarum* (black) found (barcoded specimens) . Restoration status in 2020 (stream color) and the year of restoration are indicated. Three in‐stream barriers (1 tunnel, 2 weirs) are indicated together with their date of removal in the Boye catchment (brown “x” and stream section). Catchment areas are indicated with dashed lines and catchment names are written in capitals. Furthermore, the location of the sampling area (https://www.umwelt.nrw.de/system/files/media/images/2015‐07/Karte.jpg) in Germany (https://en.m.wikipedia.org/wiki/File:Locator_map_RVR_in_Germany.svg) is shown.

The sampling sites were mainly located in the Emscher catchment (35 sites), but also in adjacent catchments (3 Ruhr, 2 Lippe, 1 Rhine sites) and were the same as in Weiss et al. (2024) (Figure 1 and Table S1). The Emscher is a right tributary to the river Rhine (catchment area 775 km^2^) and has several larger tributaries, such as the Berne (catchment area, 62 km^2^) and the Boye (catchment area, 75 km^2^). Most of the sampling sites were located in these two catchments (23 Boye and 8 Berne catchment sites), and four sites were in other tributaries of the Emscher. The chosen catchments differ in their size and in the progress of the restoration, with most of the larger Boye catchment already restored, while parts of the smaller Berne catchment still contain wastewater (Figure 1). All sites north of the Emscher were abbreviated with BO, and the sites south of the Emscher were abbreviated with BE. The notation of two sites in one stream is indicated by a “&” symbol between the individual sites in the text.

In total, we visited 41 sampling sites in March and April in both 2019 and 2020. The focal species, *G. pulex* and *G*. *fossarum*, were not found at all sites and not always at both time points (Table S1). While we found both species at 14 of these sites in sympatry, *G. pulex* was exclusively found at 20 sites and *G*. *fossarum* at six sites (Figure 1 and Table S1). For some of the restored Boye sites, we had data when both species were first found after restoration (Gillmann et al. 2023). In 2019, we aimed at sampling 5 specimens per site and species, while we increased sampling effort in 2020, aiming at 10 specimens per site. In both years, the organisms were sampled using sieves and kick‐nets, preserved in 96% ethanol, and stored at 4°C until further processing.

### 2.2 Genotyping

Prior to DNA extraction, specimens were morphologically identified using the identification key of Eiseler (2010). In cases where morphological identification was inconclusive, we included up to 5 (2019) or 10 (2020) additional individuals per site for DNA barcoding. Depending on sampling success and species identification, we extracted the DNA of 1–20 specimens per site (Table S1). In 2019, DNA was extracted following a salt precipitation protocol (Weiss and Leese 2016). In 2020, pipetting steps of the extraction were conducted on a Biomek FX^P^ liquid handling workstation (Beckmann Coulter, Bread, CA, USA) using a modified version of the bead extraction protocol of the NucleoMag Tissue kit (Macherey‐Nagel, Düren, Germany) as described in Buchner et al. (2021). After the extraction, DNA concentration was measured with the Qubit dsDNA BR Assay Kit (Life Technologies; Thermo Fisher Scientific). For all specimens, the barcoding fragment of the mitochondrial cytochrome c oxidase I (COI) gene was amplified with the primer pair LCO1490‐JJ/HCO2198‐JJ (Astrin and Stüben 2008). For both years, we used similar protocols for PCR and clean‐up as described in Weiss et al. (2024), except that for the 2019 samples, the annealing temperature in the PCR was 50°C. Bidirectional Sanger sequencing was performed at Eurofins Genomics.

Samples for ddRAD‐seq were chosen based on location, year, COI haplotype, and DNA concentration, aiming to analyze 4 to 5 specimens per site and species in 2019 and eight in 2020, if enough individuals of the respective species had been found at the respective site. In total, we sequenced 591 individuals of both species on 8 sequencing lanes. The first two lanes comprised most of the specimens from 2019, while lanes 3–6 contained specimens from 2020. On lanes 7 and 8, which mainly contained specimens of *P. coxalis* or *Sialis lutaria* (see Weiss et al. 2024; Weiss and Leese 2025), 27 additional 2019 specimens were analyzed, of which 12 were repeated because of bad quality in the first sequencing runs. Protocols to generate the ddRAD libraries differed slightly between years, that is, for the first two lanes, different clean‐up and size selection protocols were used in comparison to the other lanes, which were generated as described in Hupało et al. (2023). For the 2020 lanes, clean‐ups and size selections were all conducted on a Biomek FX^P^ liquid handling workstation (Beckmann Coulter, Bread, CA, USA) using the NucleoMag NGS clean‐up and size select kit (Macherey‐Nagel). In 2019, all pipetting steps were conducted manually, and we used the EXTRACTME DNA clean‐up kit (BLIRT S.A.) for the clean‐ups after RNase A digestion, double digestion and ligation and the AmpureXP bead system (Beckman Coulter) for PCR clean‐up. For size selections, we used SPRIselect beads (Beckman Coulter) as described in Vendrami et al. (2017). Details of the sample preparation for each individual are given in Table S2.

### 2.3 COI Data Analysis

All COI sequences were assembled and edited in Geneious Prime 2022.0.2 (https://www.geneious.com) and species assignment was checked by comparing the sequences with the Genbank nr database (NCBI Resource Coordinators 2018). Sequences of each species were aligned with MAFFT 1.4.0 (Katoh and Standley 2013) as implemented in Geneious with default settings. Sequences that were too short or had a bad quality were excluded from further analyses (Table S2) and alignments cropped to the length of the shortest remaining sequence. All analyses were conducted separately for *G. pulex* and *G*. *fossarum*. First, minimum spanning networks (Bandelt et al. 1999) were generated using Popart v.1.7 (Leigh and Bryant 2015) and colored according to catchments. For both species, known or potential cryptic species exist which we hereafter referred to as molecular operational taxonomic units (MOTUs). Groups within known MOTUs are called haplotype groups. To name the MOTUs, COI sequences were compared to barcodes from Wattier et al. (2020) for *G*. *fossarum* and from Lagrue et al. (2014) and Grabner et al. (2015) for *G. pulex* . Spatio‐temporal differentiation between populations from different years and sampling sites was inferred from differences in haplotype and nucleotide diversity and pairwise *F*_ST_ values calculated in Arlequin (v 3.5.2.2, Excoffier and Lischer 2010) for both years separately as well as combined. Here, only sites with more than five specimens were included. *F*_ST_ values and corresponding p‐values were plotted in heat maps using RStudio (Posit team 2024) and the R packages reshape 2 (Wickham 2007) and ggplot2 (Wickham 2016). Furthermore, mean *F*_ST_ values were calculated from the pairwise values for comparisons within and between catchments. To test for isolation by distance (IBD), Mantel tests were conducted with the R package vegan (Oksanen et al. 2019) using *F*_ST_ values as genetic and the waterway between sites as geographic distances. Distances were plotted using the R graphics package (v.4.4.0; R Core Team 2024). Waterway distances were calculated using QGIS v. 2.14.14 (http://qgis.org) with a stream layer provided by the federal state authority LANUV (Gewässerstationierungskarte des Landes NRW LANUV NRW (2013)). Furthermore, maps for visualization of the haplotype distribution were generated in QGIS v.2.14.14 (http://qgis.org) and edited with Adobe illustrator 2024. To test if the genetic diversity differs significantly between populations of the Berne and the Boye catchment, a Mann–Whitney *U*‐test within the R package exactRankTests (Hothorn and Hornik 2019) was conducted for haplotype and nucleotide diversity and data visualized as boxplots with the R graphics package (v.4.4.0; R Core Team 2024).

### 2.4 ddRAD Data Analysis

Preprocessing of all ddRAD‐seq libraries, including demultiplexing and removing of PCR duplicates, was performed as in Weiss et al. (2018). For loci and genotype identification, denovo_map.pl of Stacks v.1.34 (Catchen et al. 2013) was used. To identify optimal parameter settings for each species, the Stacks pipeline was run with eight different parameter settings according to the guidelines in Paris et al. (2017) as described in Hupało et al. (2023). The further analyses were executed within workflows generated with the workflow management tool Snakemake (Köster and Rahmann 2012) similar to Weiss et al. (2018). For the subsequent analyses, we used the following general filtering settings: the maximum number of SNPs per locus was 12, of which only one was subsequently used; minor allele frequency of 1%; a locus had to be present in either 90% or 85% of the individuals (locus limit) to remain in the dataset. Evaluation of Stacks parameter settings and locus limit was performed by calculating basic population genetic statistics, such as observed heterozygosity (*H*_O_), observed gene diversity (*H*_S_), overall gene diversity (*H*_T_), and overall *F*_ST_ and *F*_IS_ using the R package hierfstat (Goudet 2005) in R v.4.4.0 (R Core Team 2024). Because basic population genetic statistics were similar among Stacks settings, the Stacks setting resulting in the largest number of loci was used for all further analyses in which specimens with > 40% missing data were excluded.

To identify genetic clusters and to compare them to COI MOTUs, principal component analyses (PCAs) (Patterson et al. 2006) were performed and individual ancestry coefficients were estimated based on sparse non‐negative matrix factorization algorithms (sNMF) (Frichot et al. 2014) using the R‐package LEA (Frichot and François 2015). In the sNMF analysis, the number of clusters was varied between one and 17 with 30 replicates and 200,000 iterations per replicate, and cross‐entropy values were compared to select the most probable number of clusters (*K*). Temporal and spatial differentiation was analyzed by calculating pairwise *F*_ST_ values (after Weir and Cockerham 1984) and significance was tested by bootstrapping over loci (1000 replicates, 0.025/0.975 confidence intervals) using the R‐package hierfstat. *F*_ST_ values were visualized in heat maps. Further mean *F*_ST_ values between and within catchments were calculated, and IBD was tested in the same way as described for the COI data. To assess and visualize directional relative migration rates, we estimated *G*_ST_ (after Nei 1973) with the divMigrate function (Sundqvist et al. 2016) of the R‐package diveRsity (Keenan et al. 2013). Here, only sites with more than five specimens were included, and only migration rates > 0.3 displayed for better clarity. Additionally, heat maps similar to the *F*_ST_ heat maps including all values were generated. Genetic diversity was estimated by calculating *H*_O_ and allelic richness (AR) for each population (for both years separately and combined) with the R‐package diveRsity, and diversity differences between populations from the Berne und Boye catchment for both diversity measures tested as described for the COI data.

## 3 Results

### 3.1 Population Structure of *Gammarus pulex*

*G. pulex* was found at nearly all sampling sites north of the Emscher (26 of 27), while it was less abundant at sites south of the Emscher (7 of 14; Table S1). We successfully generated COI sequences for 433 *G. pulex* specimens. The final COI alignment of 570 bp showed 81 variable sites, of which four were non‐synonymous substitutions. We detected 38 haplotypes (H01–H38) of which four had a frequency > 8% (H01: 28.2%, H09: 8.8%, H13: 12.7%, H27: 31.9%), while all others were present in less than 2% of the specimens, with 21 being private haplotypes (Table [Link], [Link]).

**FIGURE 2:**
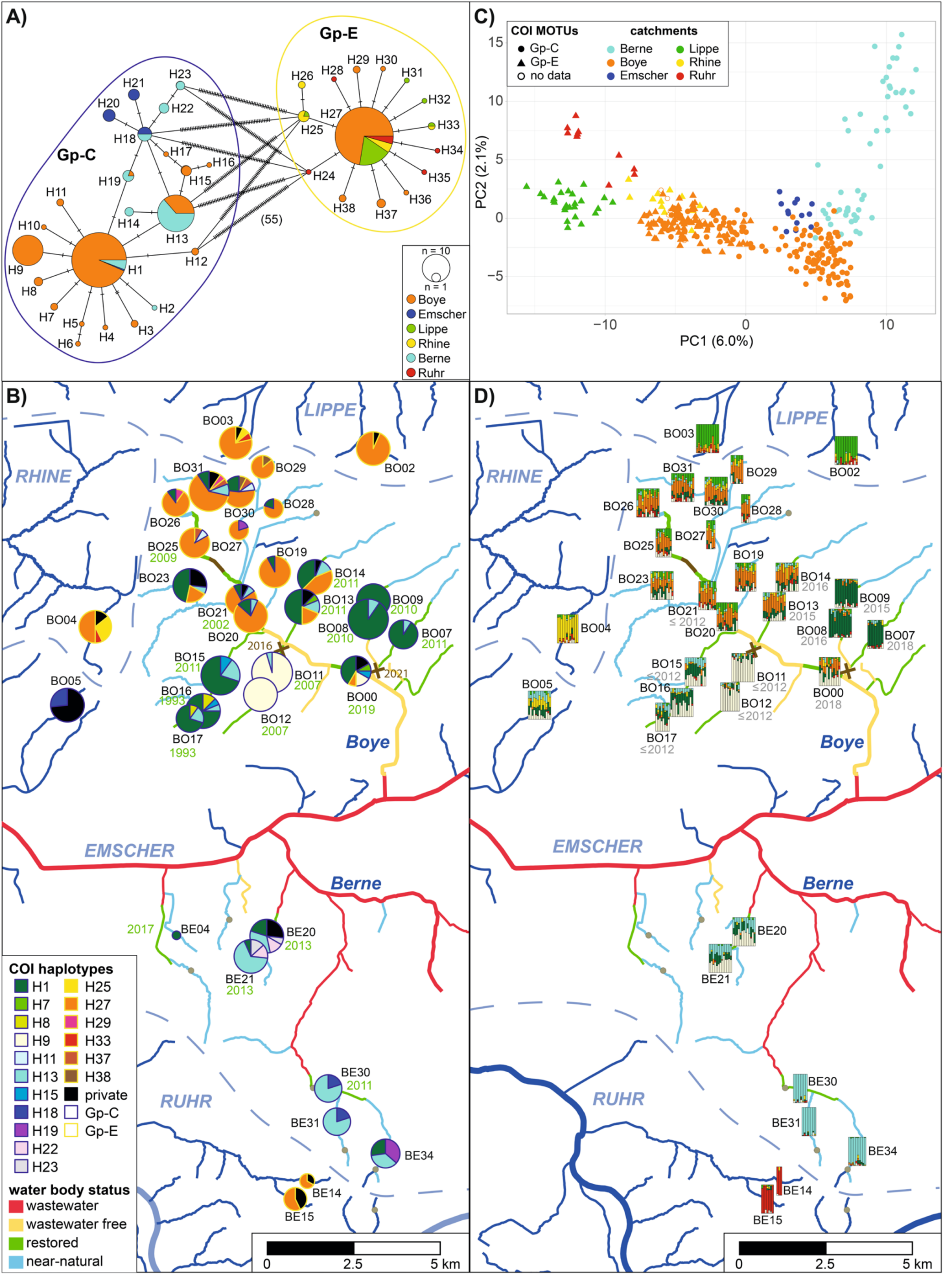
Population structure of *G. pulex* in the Emscher catchment (A) COI minimum spanning network colored according to catchments. Orthogonal dashes on network branches indicate mutations between haplotypes. (B) COI haplotype map showing the haplotype distribution. The sizes of haplotype pie charts are scaled according to the numbers of sequences per site and haplotypes of Gp‐C and E are indicated by differently colored frames. Below the names of the restored sites, the year of restoration is displayed. (C) Principal component analysis of the ddRAD‐seq data. Individuals are colored according to catchments and different symbols are used for different COI MOTUs. (D) Ancestry estimates from sNMF analysis for *k*= 7 are displayed on the map, with vertical bars representing individual ancestry coefficients. Where known, year of first occurrence is indicated below the site names.

The minimum spanning network (Figure 2A) indicated the presence of two distinct MOTUs, which could be assigned to the reported groups Gp‐C and Gp‐E (sensu Grabner et al. 2015 and Lagrue et al. 2014). Gp‐C was the more diverse group in terms of the number of haplotypes (23) and mutations between haplotypes. It contained three of the four higher frequency haplotypes (H01, H09, and H13). Haplotypes of Gp‐C and Gp‐E were differentiated by at least 55 mutations (i.e., 9.6% sequence divergence). The main haplotype of Gp‐E was H27, while the 14 other haplotypes were only found in one to five specimens and differed mostly by one mutation.

While Gp‐C was only found in the Emscher catchment, including the Berne and Boye sub‐catchments, Gp‐E was found in the Boye as well as in adjacent catchments north (Lippe, Rhine) and south (Ruhr) of the Emscher, but not in the Berne catchment (Figure 2A,B). At most of the northern Boye sites, both groups occurred in sympatry (13 sites in total), while at 15 and 6 sites, respectively, only Gp‐C or Gp‐E was found (Figure 2B). Consequently, nucleotide and haplotype diversity differed between sites, ranging from 0 to 0.053 (mean 0.014) and 0 to 0.84 with a mean of 0.43, respectively (Table S4). While the mean haplotype diversity was higher in the Berne catchment and the mean nucleotide diversity higher in the Boye catchment, these differences were not significant (Figure S1A). Most of the pairwise *F*_ST_ values (73%) indicate a significant population differentiation, including the temporal comparison for populations sampled at BE21 in 2019 and 2020 (Figure S2A). Mean *F*_ST_ values within catchments were always lower than between catchments (Table 1). However, while in some cases population differentiation within catchments was high between adjacent streams, not all comparisons of populations from different catchments were significant. Furthermore, no significant IBD pattern was detected (Figure S3A).

**TABLE 1:** Mean differentiation (*F*_ST_) for COI and ddRAD data within and between catchments for both species.

Catchment (sites)	*G. pulex*				*G. fossarum*			
	Mean *F*_ST_ COI (% significant)		Mean *F*_ST_ ddRAD (% significant)		Mean *F*_ST_ COI (% significant)		Mean *F*_ST_ ddRAD (% significant)	
	Within	Between	Within	Between	Within	Between	Within	Between
Lippe (BO02&03)	0.01 (0)	0.38 (67)	0.01 (0)	0.18 (100)	—	—	—	—
Rhine (BO04)	—	0.27 (71)	—	0.17 (100)	—	—	—	—
Emscher (BO05)	—	0.42 (100)	—	0.14 (100)	—	—	—	—
Boye (BO00, BO07–31)	0.36 (62)	0.40 (83)	0.06 (80)	0.15 (100)	0.01 (0)	0.69 (88)	0.03 (40)	0.11 (100)
Emscher (BE02)	—	—	—	—	—	0.60 (71)	—	0.06 (100)
Emscher (BE04&05)	—	—	—	—	0.15 (100)	0.49 (69)	0.01 (0)	0.07 (100)
Ruhr (BE14&15)	—	0.26 (55)	—	0.28 (100)	0.03 (0)	0.85 (100)	0.01 (0)	0.10 (100)
Ruhr (BE16)	—	—	—	—	—	0.90 (100)	—	0.10 (100)
Berne (BE20–35)	0.11 (40)	0.47 (99)	0.07 (80)	0.17 (100)	0.15 (43)	0.68 (91)	0.01 (67)	0.08 (100)

To investigate population structure with better resolution, we analyzed ddRAD data for 364 specimens. Depending on Stacks and loci filtering settings, we obtained between 198 and 663 polymorphic loci when including all specimens (Table S5). Because basic population genetic statistics were similar between settings, we further used the Stacks setting resulting in the highest number of loci and a locus filtering threshold of 85%. For the final dataset, we excluded specimens with > 40% missing data (9 specimens), resulting in 808 loci. To get first insights into the population structure and test if the two divergent mitochondrial MOTUs Gp‐C and Gp‐E represent cryptic species, we conducted a PCA. For the PCA, the first 12 axes were significant, with the first three explaining 6.0%, 2.1%, and 1.5% of the variation, respectively. In the PCA, the individuals roughly clustered according to the river catchments, despite some overlaps between them (Figure 2C). In the Boye, two ddRAD groups were detected, which were congruent with COI MOTUs. However, all specimens of these groups originated from populations where only one MOTU was found. In contrast, specimens from sympatric populations clustered indistinguishably together, indicating the occurrence of only one species in the area, despite high COI divergence.

The fine‐scale population structure is also visible in the sNMF analysis, where lowest cross‐entropy values were found for *k* = 7, but they were similarly low from *k* = 4 onwards (Figure S4A). Because geographically meaningful clusters were added from *k* = 4 to *k* = 7, we decided to display the structure for *k* = 7 in the map (Figure 2D). Here, the populations from the Ruhr catchment (BE14, BE15; red cluster) were clearly separated from all other populations, as were populations from Lippe (BO02, BO03; light green) and Rhine catchment (BO04; yellow). The other population located outside of the Berne and Boye catchment, BO05 (Emscher catchment), did not constitute a separate cluster, but was a mix of different clusters, otherwise present in Rhine, Boye, and Berne populations. The fourth cluster (i.e., turquoise) was mainly detected in three of the Berne catchment populations (BE31, BE30&34), but also to a lower proportion in the two other Berne populations (BE20&21) and the Boye catchment populations from Vorthbach (BO15&16&17). Between the latter two groups, also the fifth (i.e., white) cluster was shared, which was otherwise found as a main cluster in the two Kirchschemmsbach populations (BO11&12) and, with lower admixture proportions, in the most downstream Boye population (BO00) and some of the upper Boye populations. These upper Boye populations, including populations from the upper tributaries (i.e., BO13&14, BO19–31) and the downstream Boye population BO00, mainly share the sixth (i.e., orange) cluster. Clearly separated from all other populations were the populations BO07 and BO08&09 (i.e., dark green) from two neighboring tributaries (Wittringer Mühlenbach and Nattbach) in the downstream part of the Boye catchment.

In general, most of the *F*_ST_ values (> 90%) indicated a significant population differentiation, except for comparisons between populations within the same tributary (Figure S2B). Also, in the upper Boye region (BO20—BO31), most populations (87%) were not differentiated. However, this did not include populations from Alter Haarbach (BO19), Haarbach (BO13&14), and lower Boye (BO00), which shared higher proportions of the same cluster in the sNMF analysis but showed low, yet significant differentiation to all other populations. Most of the populations were stable over the 2 years, except BO08 and BO23, where a significant differentiation was found between years. Populations from different catchments were always significantly differentiated (values between 0.14 and 0.28; Table 1). In contrast, mean *F*_ST_ values within the Berne and Boye catchments were lower (0.07 and 0.06, respectively), with 80% of the *F*_ST_ values being significant within each catchment. The described population structure is also visible in the relative migration network, where low *F*_ST_ values corresponded to high migration rates (*G*_ST_, Figure S5).

In contrast to the COI data, a strong IBD pattern was detected (*r* = 0.62, *p* = 0.0001) even though some *F*_ST_ values from comparisons between catchments (i.e., Lippe catchment—upper Boye populations) were lower than expected (Figure S3B). Genetic diversity in terms of allelic richness (AR) ranged from 1.29 to 1.53 (mean: 1.44) with the lowest values in the Ruhr and upper Berne catchment (BE15; BE30, 31 and 34) and the highest values in the upper Boye catchment. Observed heterozygosity (*H*_O_) was more constant with values ranging from 0.12 to 0.2 (mean 0.16; Table S4). Both *H*_O_ and AR differed significantly between the Boye and Berne catchments, with higher diversity found in the Boye catchment (Figure S1B).

### 3.2 Population Structure of *Gammarus fossarum*

**FIGURE 3:**
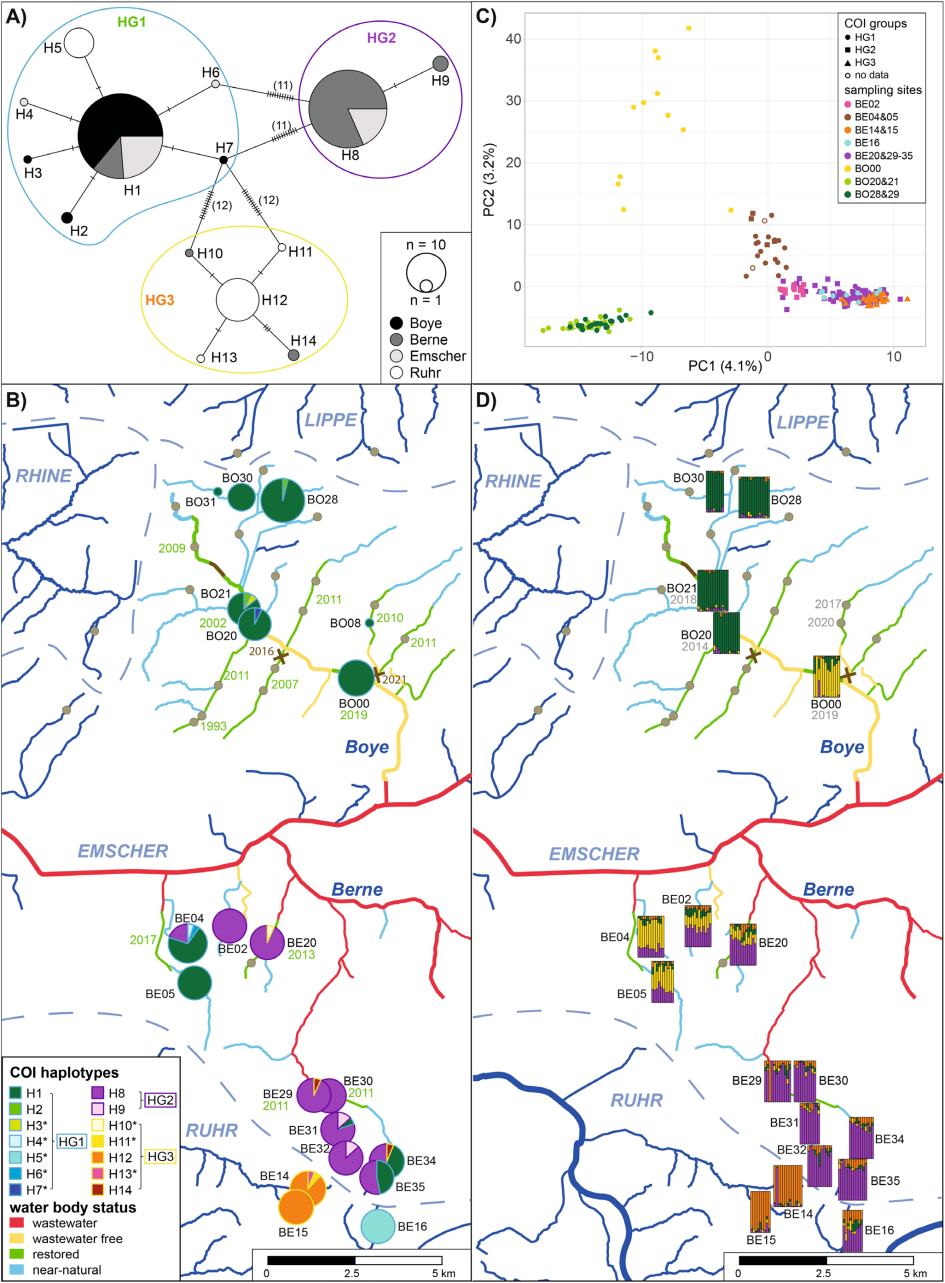
Population structure of *G. fossarum* in the Emscher catchment: (A) COI minimum spanning network colored according to catchments. Orthogonal dashes on network branches lines indicate mutations between haplotypes. (B) COI haplotype map showing the haplotype composition. The sizes of haplotype pie charts are scaled according to the numbers of sequences per site. Private haplotypes are indicated by an asterisk. Below the names of the restored sites, the year of restoration is displayed. (C) Principal component analysis of the ddRAD‐seq data. Individuals are colored according to location and different symbols are used for different COI haplotype groups. (D) Ancestry estimates from sNMF analysis for *k* = 4 are displayed on the map, with vertical bars representing individual ancestry coefficients. Where known, year of first occurrence is indicated below the site names.

In contrast to *G. pulex* , *G*. *fossarum* was found mostly south of the Emscher (14 of 15 sites). North of the Emscher, it was only detected at seven of the 27 sites, all belonging to the Boye catchment (at two sites only 1 specimen; Table S1). We obtained 286 high‐quality sequences. The final alignment was 534 bp in length and contained 30 variable sites, which were all synonymous substitutions. In contrast to *G. pulex* , we detected only 14 haplotypes, which can be divided into three groups (HG1–3) and are differentiated by at least 11 mutations (Figure 3A). Comparing sequences of these groups to known cryptic species revealed that all belong to the diverse *G*. *fossarum* ABGD‐MOTU 2, which is congruent with the originally described type B (Müller 2000; Wattier et al. 2020). All haplotype groups consisted of one main haplotype each (H1, H8, and H12); detected in > 10% of the specimens; and six, one, and four further haplotypes, and were found with one exception (H5, 5.2%) in < 2% of the specimens (Figure 3A and Table S6).

Of the 14 haplotypes, eight were exclusive to one sampling site, that is, private haplotypes. While HG1 was detected in all four catchments, HG2 only occurred in the Berne catchment and the two other streams of the Emscher catchment (Figure 3A,B). HG3 was mainly found at the two sites of the Ruhr catchment, but also in three specimens of the Berne catchment. Similar to *G. pulex* , differentiation between sampling sites was generally high, with 75% of the *F*_ST_ values indicating significant differentiation (Figure S2C). The strongest differentiation was found between populations from the Ruhr catchment (BE14 and BE15: mean *F*_ST_ 0.85 and BE16: 0.90; summarized in Table 1) and all other sites. Furthermore, populations from the Berne and Boye catchments were mostly significantly differentiated (mean *F*_ST_ 0.68, 91% significant and 0.69, 88% significant, respectively) with the exception of BE04 and BE05 (both southern tributaries to the Emscher), which were not differentiated from the Boye but from the Berne populations. While the Boye populations were not differentiated from each other, differentiation was detected between some of the Berne populations (mean *F*_ST_ 0.15, 43% significant), separated by only a few kilometers. Furthermore, none of the temporal comparisons indicated significant differentiation.

In general, mean *F*_ST_ values between catchments and within the Berne but not within the Boye catchment were higher than for *G. pulex* , and a significant IBD pattern was detected (*r* = 0.61, *p* = 0.0001). However, differentiation across short distances was already very high in some cases (Figure S3C). Additional to an overall lower number of haplotypes, haplotype and nucleotide diversity per sampling site were also lower for *G*. *fossarum* than for *G. pulex* , with nucleotide and haplotype diversity ranging from 0–0.015 (mean 0.003) and 0–0.59 (with a mean of 0.18), respectively. While nucleotide diversity was significantly higher in the Berne catchment, haplotype diversity was not significantly different between both catchments (Figure S1C).

For fine‐scale population structure analysis, we analyzed ddRAD data for 227 specimens. When all specimens were included, we obtained 102–875 loci depending on Stacks and loci filtering settings. As basic population genetic statistics were similar between Stacks settings, we used the setting resulting in the highest number of loci for the final analysis (Table S5). By excluding specimens with > 40% missing loci (24 specimens), the number of loci shared between > 85% of the individuals could be increased to 2135.

To check if the mitochondrial haplotype groups were congruent with nuclear ddRAD clusters, we conducted a PCA. Here, the first 16 axes were significant, with the first three explaining 4.1%, 3.2%, and 1.9% of the variation, respectively. In the PCA, specimens from different mitochondrial groups clustered together, and differentiation was driven by geographical location rather than by haplotype groups (Figure 3C).

This is also visible in the sNMF analysis, in which four clusters were found to best represent the population structure according to the cross‐entropy criterion (Figure S4B). While the Ruhr catchment populations clustered together with the Berne populations in the PCA, two of the populations (BE14 and BE15) were clearly separated in the sNMF analysis (Figure 3D), sharing one of the clusters (i.e., orange). However, the third population from the Ruhr catchment (BE16; waterway distance to BE15 24.7 km) shared higher membership proportions with the populations from Borbecker Mühlenbach and Kesselbach (upper Berne catchment, BE29–35; waterway distance > 79 km, lowest air distance 1.17 km), which were together differentiated from all other populations (i.e., purple). The other population from the Berne catchment (BE20) shared the main cluster present in the Berne (i.e., purple), but also the cluster otherwise found in two other southern tributaries of the Emscher (sites BE02, BE04, BE05; i.e., yellow). The main cluster from the BE04 population (i.e., yellow) was likewise mainly found in the Boye population BO00, which was the most recently restored site. Interestingly, BO00 was strongly differentiated from the remaining Boye catchment populations (BO20&21, BO28, and BO30), which all shared the fourth cluster (i.e., green).

The differentiation of Berne and Boye populations is also reflected in the PCA (Figure 3C). Here, specimens from BO00 were clearly separated from Boye but also the southern Emscher populations (BE04 and BE05). While the membership proportions were in most cases similar between individuals sampled at one site, two specimens, one from BE14, and one from BE29, each only contained the cluster of the respective other site, yet each showed a site‐specific haplotype.

Similar to *G. pulex* , most of the pairwise comparisons (> 89%) indicated a significant population structure, but differentiation was in contrast to the COI data generally lower than in *G. pulex* (Figure S2D). While in the Boye system only BO00 was differentiated from all other populations, low but significant differentiation was detected in some cases in the upper Berne system between BE34 and BE35 and sites BE29–32, except for the geographically closest sites BE30 and BE34. Most of the populations were not differentiated among years, except for populations BE04, BE05, and BO28 (Figure S2D). Similar to *G. pulex*, all comparisons between catchments were significant with mean *F*_ST_ values ranging between 0.06 and 0.11, while within catchments, differentiation was lower (mean *F*_ST_ between 0.01 and 0.03) and in less cases significant (40% of comparisons in Boye and 67% in Berne catchment, summarized in Table 1).

The described population structure is also visible in the relative migration network in which BO00 is placed between Berne and Boye populations, with gene flow directed from both catchments similarly toward this population (Figure S6). Otherwise, higher migration rates were only detected within catchments. Similar to the COI data, a strong IBD pattern was found (*r* = 0.554, *p* = 0.0001; Figure S3D).

Allelic richness was similar to *G. pulex* , ranging from 1.37 to 1.56 (mean: 1.45) with highest values at BO00, while *H*_O_ was slightly lower and ranged from 0.11 to 0.16 (mean: 0.14, Table S4). Between Berne and Boye catchments, no significant differences in genetic diversity were found (Figure S1D).

## 4 Discussion

In this study, we analyzed the small‐scale metapopulation structure of two amphipod species in a restored river system in a strongly urbanized area to identify dispersal barriers and recolonization pathways. Moreover, we combined the classical COI barcoding gene marker with high‐throughput nuclear SNP data to test if evolutionarily divergent cryptic species are present in the area.

### 4.1 No Evidence for Cryptic Species

We found different mitochondrial MOTUs or haplotype groups for both species, which were assigned to the potential cryptic species Gp‐C and Gp‐E (Grabner et al. 2015; Lagrue et al. 2014) for *G. pulex* and the diverse ABGD‐MOTU 2 or type B (Wattier et al. 2020) for *G*. *fossarum*. However, the nuclear SNP data showed that, in both cases, only one species was present in the area. For *G*. *fossarum*, this was in accordance with the expectations from literature even though *G*. *fossarum* represents a highly diverse species complex (e.g., Copilaş‐Ciocianu and Petrusek 2015; Lagrue et al. 2014; Wattier et al. 2020; Weiss et al. 2014), in which some of the cryptic species were verified with additional nuclear markers and behavioral and ecological differences have been found (e.g., Copilaş‐Ciocianu and Petrusek 2015; Eisenring et al. 2016). In contrast, fewer studies on the *G. pulex* species complex have been conducted. When analyzing mate discrimination within the species complexes of *G. pulex* and *G*. *fossarum*, Lagrue et al. (2014) found that successful mating was possible in the lab for MOTUs differing by 16% genetic distance, even though pre‐copulatory formation was rare for this genetic distance in nature. They concluded that the *G. pulex* MOTUs (Gp‐A, B, C and D; up to 11.4% K2p‐distance) should not be considered as distinct (sub)species. This is in accordance with our data for Gp‐C and Gp‐E, which were equally divergent to each other (10.8% K2p‐distance), but showed no evidence for evolutionary divergence when nuclear SNP data of sympatric specimens belonging to the different MOTUs were studied.

In addition to this mito‐nuclear discordance pattern, other discrepancies were noted for both species when comparing their population structure inferred from the two genetic marker systems. Specifically, in several instances, the COI data indicated either low or high levels of differentiation, whereas the nuclear data showed the opposite result. A previous study examining COI and microsatellite data had also identified mito‐nuclear discordance in the population structure of *G*. *fossarum* (Weiss and Leese 2016). However, in this case, the patterns were more in line with one another, as COI gene differentiation was matched by corresponding nuclear data, similar to patterns observed with ddRAD data from Weiss et al. (2022). However, in both studies, haplotypes shared between populations across larger geographic distances did not indicate contemporary gene flow, as the nuclear markers indicated strong differentiation between those sites. These findings in combination with the results from the present study highlight the importance of integrating nuclear genetic markers and that solely analyzing mitochondrial markers is not sufficient for analyzing the population structure as suggested by, for example, Schneeweiss et al. (2023).

### 4.2 Population Structure of *G. pulex*

In accordance with our first hypothesis, we found a significant differentiation of populations originating from different (sub‐)catchments. However, differentiation was not always as strong as expected and, in some cases, even lower than within catchments. According to the Stream Hierarchy Model (Meffe and Vrijenhoek 1988), population differentiation between catchments is expected for a hololimnic species like *G. pulex* , which is confined to aquatic habitats throughout its life cycle. However, gene flow across catchment boundaries has been suggested to be possible for amphipod species by active migration through underground connections in times of drought (Weigand et al. 2020) or by passive dispersal via birds (Figuerola and Green 2002; Rachalewski et al. 2013), large mammals (Peck 1975; Vanschoenwinkel et al. 2008), or humans (Westram et al. 2011).

The populations we found to be most isolated based on the ddRAD data were populations from the Ruhr catchment. Here, no indication for an overland connection to the geographically close Berne catchment was found. Interestingly, the lowest differentiation in the nuclear ddRAD data was found in comparison to the Lippe populations (BO02 and BO03), hinting toward a common ancestral gene pool during initial colonization. Also, for the northern Emscher tributary population (Alsbach, BO05), no indication of overland dispersal was found, as strong differentiation from all other populations was indicated by both genetic markers. In contrast, populations from the Lippe and Rhine catchments were mostly not differentiated from the closest Boye populations in the COI data. Also, in the nuclear data, differentiation was lower than expected, but differences in cluster membership proportions (sNMF analysis) indicated that gene flow did not happen recently nor frequently. Similarities could either be explained by past overland dispersal events or migration via the waterway prior to the construction of barriers. As expected, the still strongly modified Emscher was found to pose a strong dispersal barrier between the three Emscher subcatchments Hexbach, Boye, and Berne.

Also, in agreement with our first hypothesis, genetic diversity was significantly higher within the Boye than within the Berne catchment. The population structure within catchments could mainly be explained by recent recolonization, wastewater, and artificial in‐stream barriers. Within the Boye catchment, both mitochondrial and nuclear data indicated a division into a highly connected upper part and a strongly differentiated lower part, divided roughly by the restoration in the Boye. The upper part had been restored earlier (2002/2009), while the lower part contained wastewater until 2017 and had been only partly restored in the sampling years 2019/2020, separating right and left tributaries in this part. In contrast to *G. pulex* , upper Boye populations from *A. aquaticus* and *P. coxalis* were more strongly isolated (Weiss et al. 2024). This indicates that the population structure is shaped by different species‐specific characteristics, but probably also by the competition between both species groups (Gillmann et al. 2024) as both have a similar ecological niche (Graça et al. 1994). In comparison to the isopods, *G. pulex* exhibits greater mobility and dispersal capabilities, explaining the higher connectivity. Additionally, a relatively recent colonization of the restored sites in the upper Boye part might have led to the low population structure. Furthermore, several streams or stream sections in the upper Boye catchment are temporary streams, which need to be repeatedly recolonized after droughts, enhancing genetic homogeneity. In the upper part, few large in‐stream barriers exist, except one pumping station between BO25 and BO21. Here, the water of the Boye is pumped through a pipe due to mining subsidence, being unpassable for hololimnic species. However, against our expectations, the upstream populations BO25&26 were not genetically isolated. Therefore, we conducted more research and found that a bypass exists between the Wiesentalbach (BO30&31) and the Boye upstream of the pumping station (personal communications Untere Landschaftsbehörde), which seemingly can be used by *G. pulex* for migration between streams.

While in the upper part of the Boye catchment high connectivity was found over up to 8.2 km, most tributaries in the lower part were isolated from each other already at shorter waterway distances (lowest distance 3 km). This isolation can mostly be explained by the wastewater conditions in the downstream part of the Boye. However, we found one indication (BO13&14) that migration had even been possible through the wastewater, but that after colonization, gene flow was reduced. The most recently restored site in the Boye (BO00) was probably recolonized after wastewater removal from different upstream populations, which then intermixed, leading to a significant population differentiation at this site, even though *G. pulex* was sampled only one year after its first detection in 2018.

In contrast, the other restored sites were not recolonized from upper Boye populations, but probably from near‐natural upstream parts of the respective streams. Two of the streams had been separated by weirs, which were removed in 2016 (BO11&12) and 2021 (BO07–09). Despite the removal of wastewater (BO15–17) and the barrier (BO11&12), populations from these streams remained isolated. Such an isolation pattern has also been found for *A. aquaticus* (Weiss et al. 2024). Interestingly, these populations were in both cases more closely related to some of the Berne populations than to the upper Boye catchment populations. However, we cannot infer the direction of dispersal from our results, but the genetic similarity indicates that passive transport via birds or humans might have played an important role for recolonization. The persisting isolation in the absence of a permanent barrier might indicate restricted gene flow, despite possible migration routes. Possible explanations are either a density‐dependent priority effect (Fraser et al. 2014), or a combination of numerical advantage together with adaptation of the first migrants (monopolization hypothesis, De Meester et al. 2002), which has been found for other species (e.g., Funk et al. 2011; Nosil et al. 2009; Nosil et al. 2008; Urban and De Meester 2009).

Within the Berne system, two groups were found, which were clearly separated by the stream sections containing still wastewater. Whereas no differentiation was detected between BE30 and BE31 (distance 1.29 km), the other population in the Borbecker Mühlenbach (BE34, distance 3 and 3.8 km, respectively) was already significantly differentiated from both sites. In contrast, within the Boye system, no differentiation was found over more than 8 km, indicating that distance alone cannot explain the population structure.

In summary, the population structure we detected at the small scale of the study area was stronger than expected in comparison to other population genetic studies of *G. pulex* , where low genetic variation was found over larger distances and mostly only between streams (Müller 1998; Švara et al. 2019; Weigand et al. 2020). In some cases, a significant isolation by distance was found within streams, yet mostly at larger distances, and in one river, no differentiation was detected over 40 km (Švara et al. 2022; Švara et al. 2021). Although we detected a strong overall population structure with both genetic markers, the detailed pattern differed, and the fine scale population structure could only be resolved with the nuclear SNP data. In other studies, COI sequences, allozymes, and microsatellites were used, which have been found to be less suitable to detect fine‐scale differences (Rašić et al. 2014; Vendrami et al. 2017). This could explain why we found a stronger population structure within *G. pulex* than the other studies. However, also the strong anthropogenic impact in the study area, with isolation of populations by wastewater and in‐stream barriers, likely contributes to the comparably strong structure.

### 4.3 Population Structure of *G*. *fossarum*

In our second hypothesis, we postulated that the drivers of the population structure of *G*. *fossarum* will be similar to *G. pulex* , with an isolation of different (sub‐)catchments and isolation by wastewater and in‐stream barriers within catchments, but that the differentiation will be stronger and genetic diversity lower for *G*. *fossarum* due to its higher sensitivity to environmental stressors. Overall, we found a similar population structure, with wastewater being a strong driver of isolation. The fact that we found no, or only a few, *G*. *fossarum* specimens at sites above the in‐stream barriers analyzed for *G. pulex* might already indicate that these prevented recolonization of the respective tributaries or stream parts for *G. fossarum*. However, the barrier effects could not be analyzed directly, and other factors such as interspecific competition with *G. pulex* or the isopods *A. aquaticus* and *P. coxalis* as well as stressor sensitivity could have shaped the distribution.

In accordance with the hypothesis, we found a higher differentiation and lower diversity in the mitochondrial COI data in comparison to *G. pulex* . However, considering the nuclear data, overall differentiation was lower and diversity similar. The detected differentiation was also lower and mean allelic richness higher than in another anthropogenically impacted, but less urbanized area (Sauerland, Ruhr‐catchment), where the population structure of *G*. *fossarum* had been analyzed with the same methods (Weiss et al. 2022), but mostly over larger distances. The genetic diversity of the two amphipod species in terms of allelic richness was comparable with the more pollution tolerant isopod species analyzed in the same area (Weiss et al. 2024), while mean observed heterozygosity was lowest for *A. aquaticus* (0.12), highest for *G. pulex* (0.16) and intermediate for *G*. *fossarum* and *P. coxalis* (0.14). These results indicate that it is not possible to directly infer the level of genetic diversity and differentiation from general sensitivity to stressors or from the level of urbanization or overall stream modification, highlighting the importance of empirical studies.

While the overall population structure of *G*. *fossarum* resembled that of *G. pulex*, the detailed population structure differed. Within the Ruhr catchment, *G*. *fossarum* was found at three sites belonging to two different sub‐catchments (i.e., Ruhmbach BE14 and BE15 and Wolfsbach BE16), which were divided by a stream distance of 24.7 km and significantly differentiated from each other. However, the differentiation to the Berne catchment populations with low air distance was not as strong as for *G. pulex* based on the nuclear data. While specimens from Ruhmbach, Wolfsbach, and upper Berne catchment clustered together in the PCA, differentiation was visible in the sNMF, especially for the Ruhmbach catchment. In contrast, no similarities between these populations were found in the COI data, where both Ruhr subcatchments were strongly differentiated from all other streams containing only private haplotypes. One explanation for the lower differentiation between Ruhr and Berne catchments in the nuclear compared to the mitochondrial data could be past but not ongoing vector‐mediated overland dispersal followed by the fixation of different haplotypes due to genetic drift. Haplotype H1, which had been found at BE34&35, was also frequent in a more eastern part of the Ruhr catchment (approx. 30 km air distance) (Weiss and Leese 2016). Therefore, it is possible that it was previously also present at BE16 or still is in low frequency and was not detected in this study due to sampling bias. Within the nuclear data, we found one specimen at BE29 and BE14 (both 2019), each belonging to the opposite cluster. This could indicate a rather recent exchange but more likely resulted from a mistake during lab work given that no similar exchange was found in the mitochondrial data.

In contrast, populations from the Boye catchment were strongly differentiated from all other catchments, as were populations from the other southern Emscher tributaries, indicating the barrier effect of the Emscher. However, the isolation was not as strong as expected, except for the upper Berne catchment. The similarities we detected, especially between the neighboring tributaries (BE02 and BE20) suggest relatively recent overland dispersal, as was also found for *G. pulex* at several locations.

Unlike for *G. pulex* , we did not expect significant genetic diversity differences between Boye and Berne populations because *G*. *fossarum* was much less abundant in the Boye catchment and, in contrast, more abundant in the Berne catchment, which could lead to similar metapopulation sizes and a similar genetic diversity. Only nucleotide diversity was higher in the Berne catchment, while the other diversity measures did not differ significantly. Even though the Berne catchment is still more strongly modified, the more sensitive species *G*. *fossarum* was more abundant here. Alonso et al. (2010) found that *G*. *fossarum* can be more tolerant to certain stressors than *G. pulex* , and other studies found that sensitivity to certain stressors can also differ within both species (Adam et al. 2010; Schneeweiss et al. 2023). Therefore, in addition to competition with *G. pulex* , which dominated in the Boye catchment, adaptation to different stressors and priority effects might have played a role in shaping the distribution and the population structure of both species.

Within the Berne catchment, we found a strong isolation by wastewater similar to *G. pulex* . In the upper part of the catchment, that is, in Borbecker Mühlenbach and Kesselbach, *G*. *fossarum* was found at more sites than *G. pulex* , but differentiation patterns were similar, with sites BE29–32 not differentiated over up to 2.7 km. Conversely, the upstream sites of the Borbecker Mühlenbach, BE34&35, were already significantly differentiated from all other sites (distance 3.5 km—5.3 km), except BE34–BE30, which were separated by the lowest distance (3 km). This pattern shows that populations can be already significantly differentiated over a distance of 3.5 km, similar to the findings in Weiss et al. (2022). Also, within the Boye catchment, we found a division in the upper and lower part with low differentiation in the former and high differentiation in the latter. However, the downstream population from BO00 was less differentiated from the upper populations for *G. pulex* , where recolonization likely happened from several upstream populations. In contrast, the *G*. *fossarum* population at BO00 was strongly differentiated from all other populations, especially visible in the PCA and closest related to populations from a southern Emscher tributary (BE04&05). *G*. *fossarum* was first detected at this site in our first sampling year (2019) and in case of a recent recolonization from BE04 and BE05, no significant differentiation would be expected. Therefore, the upstream Boye populations are unlikely to serve as source populations for recolonization as for *G. pulex* , indicating that the Boye in the lower part still represents a migration barrier for *G*. *fossarum*. In contrast, we found no differentiation between the two populations from the near‐natural sites (BO28 and BO30) and the two restored sites in the upper Boye catchment (BO20&21), which were separated by 4.3 km waterway distance. At the restored sites, *G*. *fossarum* was first recorded in 2017/2018, indicating a relatively recent recolonization from the near‐natural upstream sites and therefore does not necessarily show that gene flow occurs frequently over this distance.

In summary, even though the differentiation between populations was lower than expected in comparison to *G. pulex* , populations originating from different streams were mostly significantly differentiated, while also in some cases reduced within streams over distances < 3 km.

## 5 Conclusions

In accordance with our hypotheses, populations from both species were isolated between (sub‐)catchments, even though evidence for overland dispersal was found in several cases. Within catchments, large in‐stream barriers and sites of wastewater influx explained many aspects of the observed metapopulation structure. Yet, not all aspects were in line with these factors, indicating that other factors, such as priority effects, adaptation, and competition could be important evolutionary drivers leading to the observed metapopulation structure. Against our expectations, the generally more stressor‐sensitive species, *G*. *fossarum* type B, did not show a higher differentiation or lower genetic diversity than *G. pulex* . This emphasizes that it is not possible to infer the level of genetic diversity and differentiation from general sensitivity to stressors, but that it must be assessed directly. From an evolutionary perspective, this study shows that a high local genetic diversity has been maintained in headwater sections in the strongly urbanized Emscher catchment. These habitats serve as crucial refugia for populations that can recolonize stream sections following restoration, highlighting the importance of connected landscape mosaics that support local biodiversity.

## Author Contributions

**Martina Weiss:** conceptualization (equal), data curation (lead), formal analysis (lead), funding acquisition (supporting), investigation (lead), methodology (lead), project administration (supporting), visualization (lead), writing – original draft (lead), writing – review and editing (lead). **Marie V. Brasseur:** conceptualization (supporting), investigation (supporting), writing – review and editing (supporting). **Armin W. Lorenz:** conceptualization (supporting), investigation (supporting), writing – review and editing (supporting). **Florian Leese:** conceptualization (equal), data curation (supporting), funding acquisition (lead), investigation (supporting), investigation (supporting), methodology (supporting), methodology (supporting), project administration (lead), project administration (lead), writing – original draft (supporting), writing – original draft (supporting), writing – review and editing (supporting), writing – review and editing (supporting).

## Conflicts of Interest

The authors declare no conflicts of interest.

## Supporting information


Figure S1.

Figure S2.

Figure S3.

Figure S4.

Figure S5.

Figure S6.

Table S1.

Table S2.

Table S3.

Table S4.

Table S5.

Table S6.

## Data Availability

The data that support the findings of this study are available under the NCBI BioProject PRJNA1163041. Additionally, demultiplexed ddRAD data used for Stacks clustering are uploaded as BioSamples, and individual accession numbers are given in Table S2. COI haplotype sequences are available under the NCBI accession numbers PQ285656–PQ285707, indicated in Table S2.
