# Supplementary material for: Population Genomics Reveals Small‐Scale Metapopulation Structure of Two Strictly Aquatic Keystone Species in a Recently Restored Urban River System (Emscher, Germany)
Source: Ecol Evol. 2025 Apr 24;15(4):e71214. doi: 10.1002/ece3.71214 (PMC12022002; doi:10.1002/ece3.71214)
Supplement: Supplementary file 1 — Figure S1. [file ECE3-15-e71214-s002.pdf]

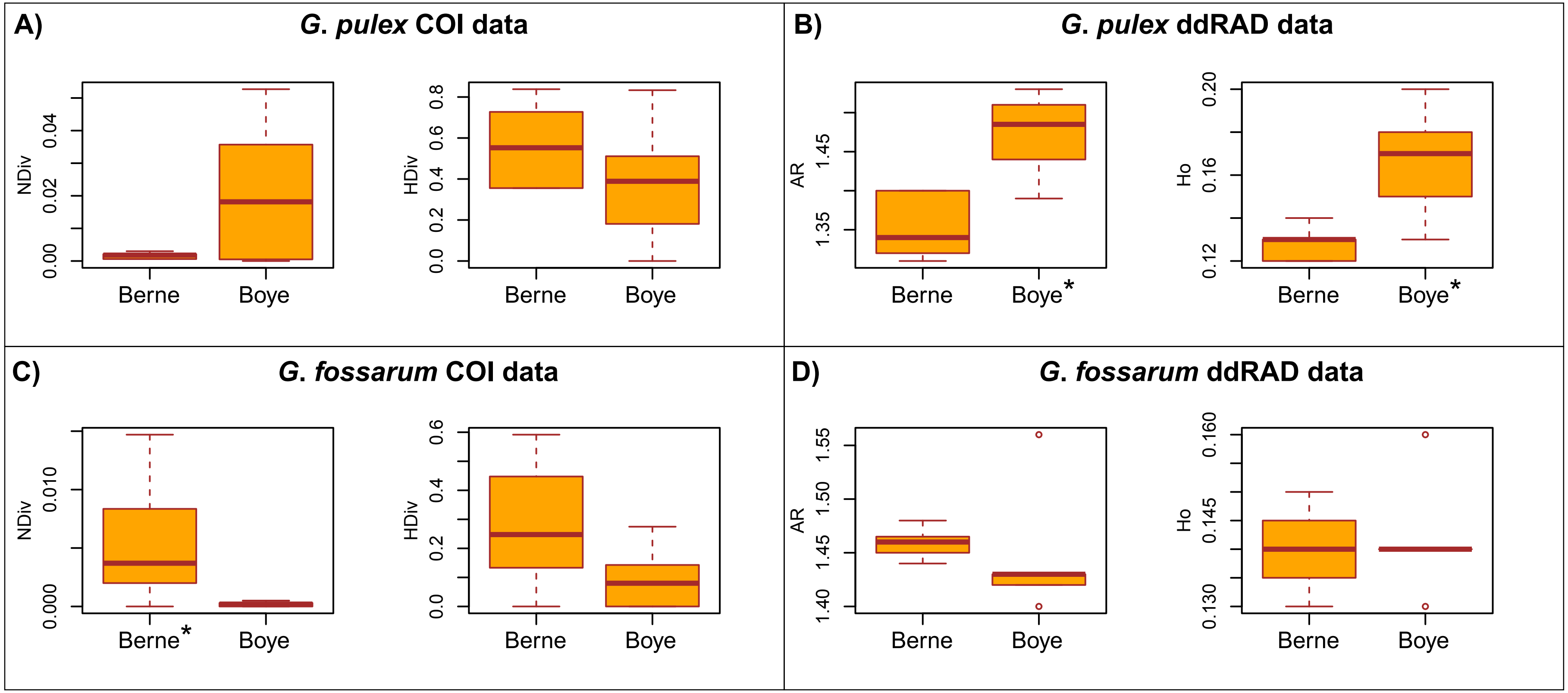

Figure S1: Comparison of genetic diversity for *G. pulex* (A, B) and *G. fossarum* (C, D) for the COI data (A, C; NDiv = nucleotide diversity; HDiv = haplotype diversity) and ddRAD data (B, D; AR = allelic richness, Ho = observed heterozygosity). If diversity measures differ significantly between catchments, the catchment with the higher diversity is indicated by an asterisk.
