# Supplementary material for: Population Genomics Reveals Small‐Scale Metapopulation Structure of Two Strictly Aquatic Keystone Species in a Recently Restored Urban River System (Emscher, Germany)
Source: Ecol Evol. 2025 Apr 24;15(4):e71214. doi: 10.1002/ece3.71214 (PMC12022002; doi:10.1002/ece3.71214)
Supplement: Supplementary file 2 — Figure S2. [file ECE3-15-e71214-s003.pdf]

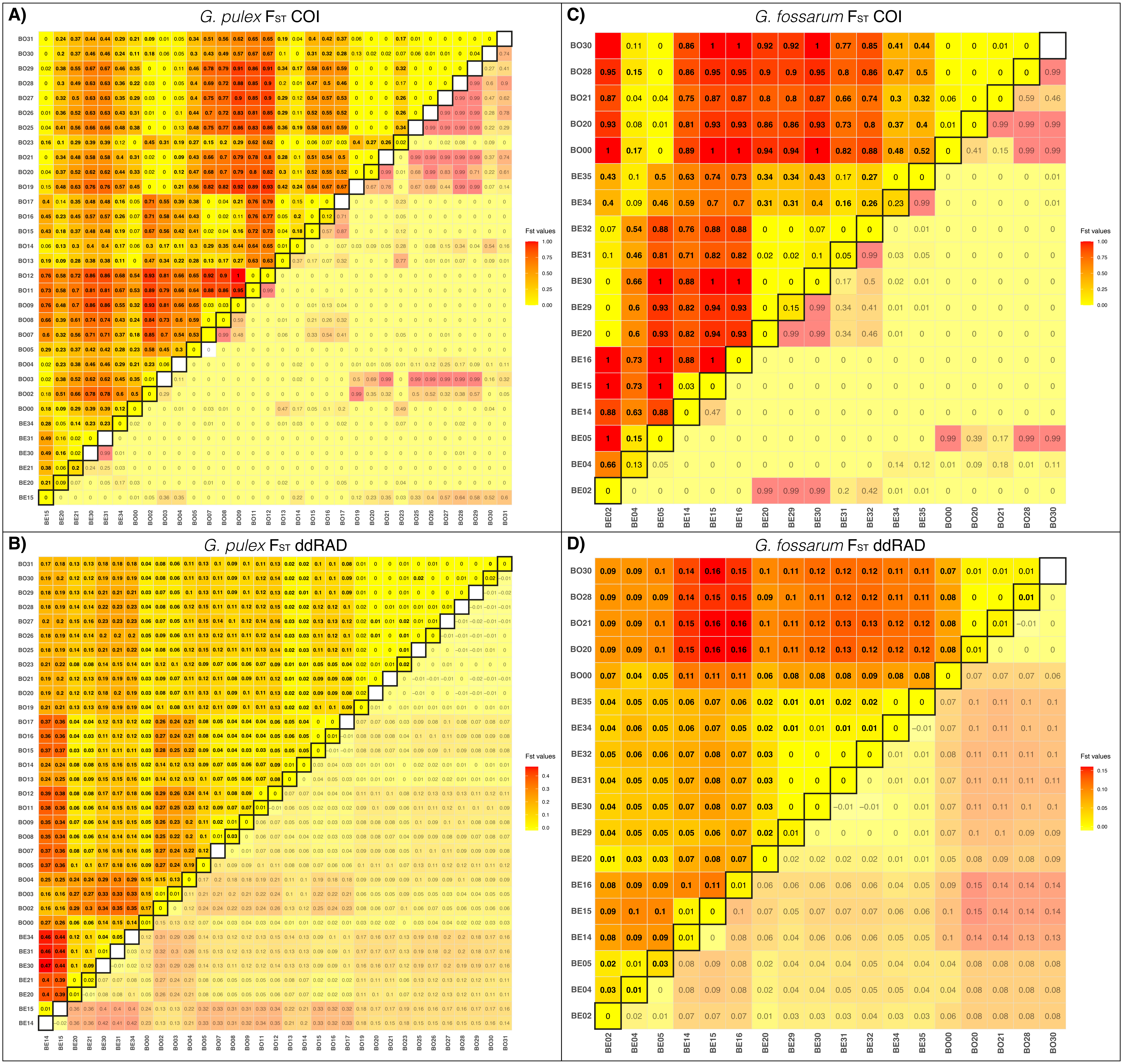

Figure S2: FST heat maps for pairwise comparisons between sampling sites for *G. pulex* (A, B) and *G. fossarum* (C, D) and COI (A, C) and ddRAD data (B, D), respectively. Above the diagonal pairwise FST values are given and below either p-values (COI data sets; values < 0.05 indicate significant differentiation) or the lower confidence interval (ddRAD data set; values > 0 indicate significant differentiation) are given. In the diagonal, FST values for the comparison between the years 2019 and 2020 are given, with a white square, when only samples from one year were available or n < 5 for one year. Significant FST values are indicated in bold and all values are colored according to the level of differentiation.
