# Supplementary material for: Population Genomics Reveals Small‐Scale Metapopulation Structure of Two Strictly Aquatic Keystone Species in a Recently Restored Urban River System (Emscher, Germany)
Source: Ecol Evol. 2025 Apr 24;15(4):e71214. doi: 10.1002/ece3.71214 (PMC12022002; doi:10.1002/ece3.71214)
Supplement: Supplementary file 3 — Figure S3. [file ECE3-15-e71214-s007.pdf]

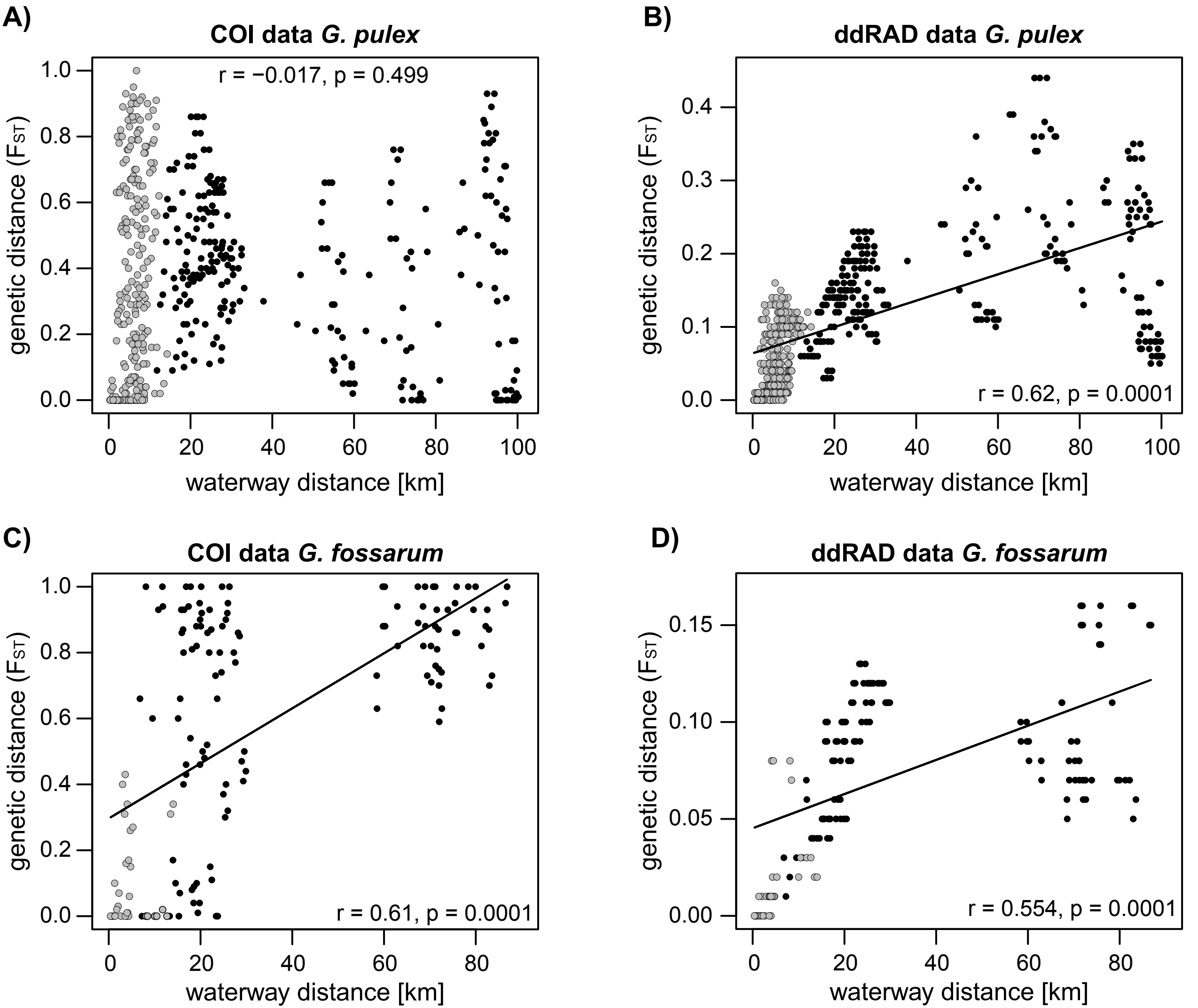

Figure S3: Correlation between pairwise genetic distances (F_{ST}; A and C COI data, B and D ddRAD data) and waterway distances for *G. pulex* (A, B) and *G. fossarum* (C, D). Distances within catchments are colored in grey and between catchments in black.
