# Supplementary material for: Population Genomics Reveals Small‐Scale Metapopulation Structure of Two Strictly Aquatic Keystone Species in a Recently Restored Urban River System (Emscher, Germany)
Source: Ecol Evol. 2025 Apr 24;15(4):e71214. doi: 10.1002/ece3.71214 (PMC12022002; doi:10.1002/ece3.71214)
Supplement: Supplementary file 4 — Figure S4. [file ECE3-15-e71214-s011.pdf]

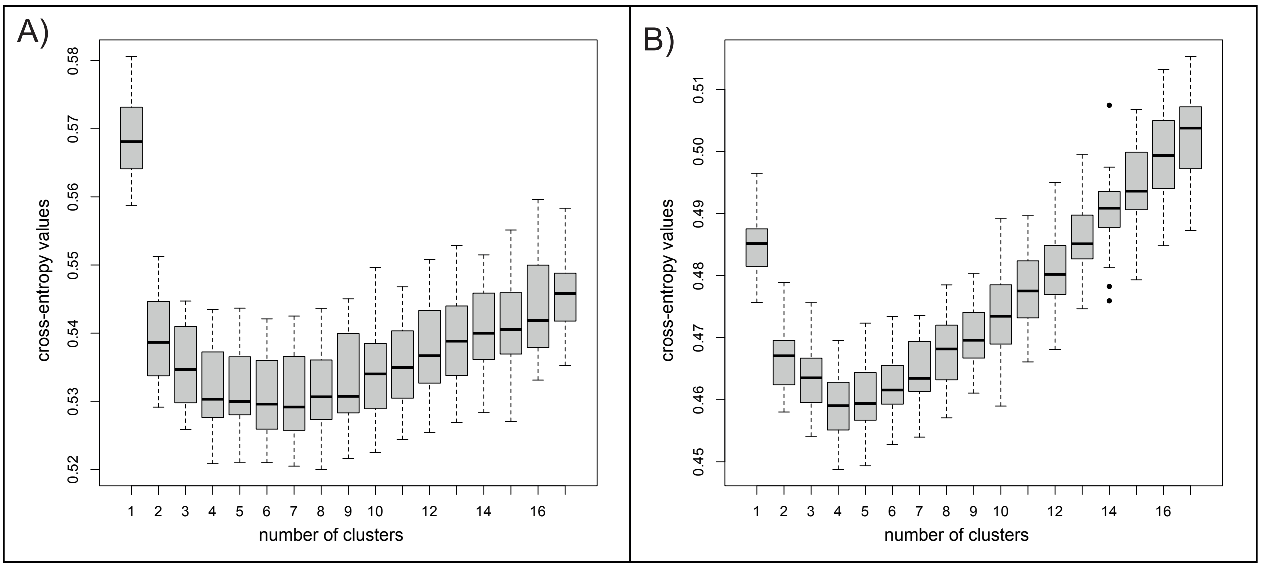

Figure S4: Standard boxplots of cross-entropy values (30 repeats) of sNMF analysis for final ddRAD datasets for A) *G. pulex* and B) *G. fossarum*, respectively.
