# Supplementary material for: Population Genomics Reveals Small‐Scale Metapopulation Structure of Two Strictly Aquatic Keystone Species in a Recently Restored Urban River System (Emscher, Germany)
Source: Ecol Evol. 2025 Apr 24;15(4):e71214. doi: 10.1002/ece3.71214 (PMC12022002; doi:10.1002/ece3.71214)
Supplement: Supplementary file 5 — Figure S5. [file ECE3-15-e71214-s008.pdf]

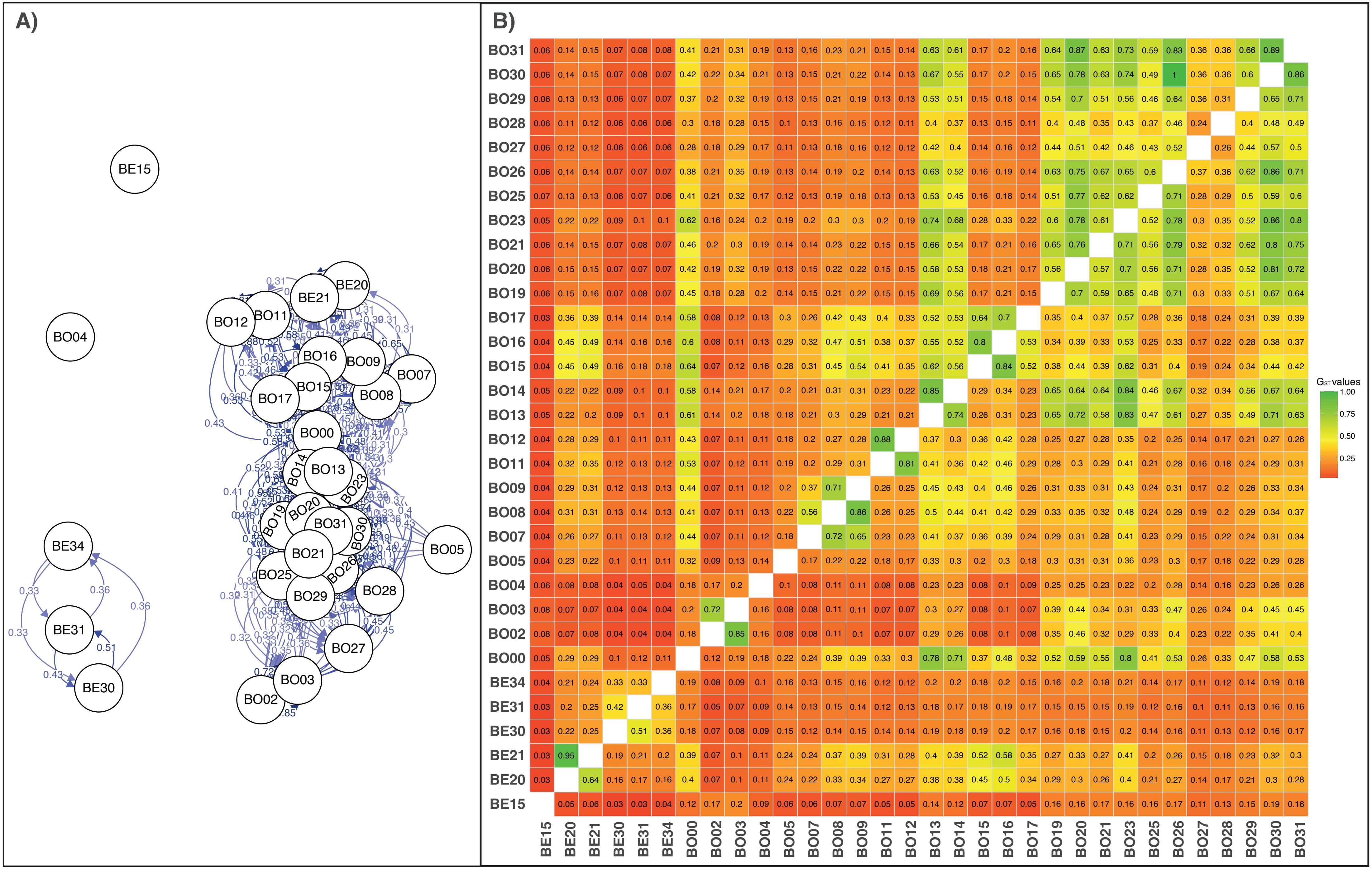


**Figure S5:** A) Relative migration network ($G_{ST}$) for *G. pulex*. Only values > 0.3 are shown. All values are shown in the heatmap (B) with rows as source and columns sink populations.
