# Supplementary material for: Population Genomics Reveals Small‐Scale Metapopulation Structure of Two Strictly Aquatic Keystone Species in a Recently Restored Urban River System (Emscher, Germany)
Source: Ecol Evol. 2025 Apr 24;15(4):e71214. doi: 10.1002/ece3.71214 (PMC12022002; doi:10.1002/ece3.71214)
Supplement: Supplementary file 6 — Figure S6. [file ECE3-15-e71214-s001.pdf]

A)

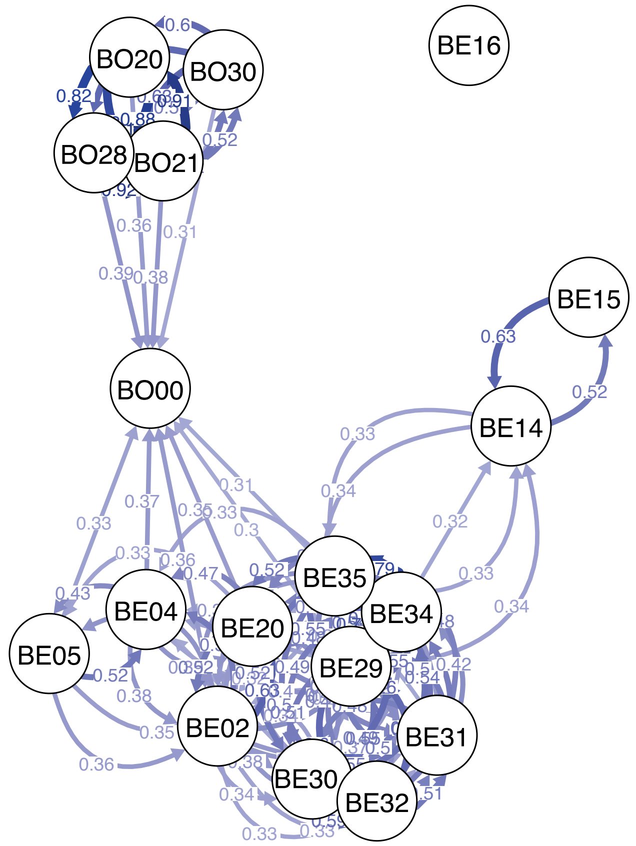

B)

	BE02	BE04	BE05	BE14	BE15	BE16	BE20	BE29	BE30	BE31	BE32	BE34	BE35	BO00	BO20	BO21	BO28	BO30
BO30	0.21	0.21	0.17	0.15	0.12	0.11	0.21	0.17	0.16	0.16	0.17	0.17	0.18	0.31	0.6	0.63	0.61	
BO28	0.25	0.26	0.21	0.17	0.13	0.11	0.25	0.2	0.18	0.17	0.18	0.2	0.21	0.39	0.88	0.92		0.52
BO21	0.23	0.24	0.19	0.16	0.13	0.11	0.24	0.18	0.16	0.17	0.17	0.19	0.19	0.38	0.91		1	0.52
BO20	0.23	0.24	0.19	0.16	0.13	0.11	0.23	0.17	0.16	0.16	0.17	0.18	0.19	0.36		0.8	0.82	0.5
BO00	0.18	0.29	0.2	0.15	0.12	0.12	0.2	0.17	0.15	0.15	0.15	0.16	0.17		0.16	0.16	0.16	0.14
BE35	0.39	0.33	0.27	0.33	0.23	0.23	0.52	0.55	0.52	0.45	0.5	0.7		0.31	0.17	0.16	0.18	0.14
BE34	0.36	0.3	0.25	0.32	0.23	0.23	0.46	0.54	0.49	0.42	0.47		0.79	0.27	0.17	0.16	0.17	0.13
BE32	0.3	0.29	0.23	0.28	0.2	0.19	0.42	0.6	0.55	0.51		0.5	0.54	0.25	0.16	0.16	0.16	0.13
BE31	0.31	0.27	0.21	0.25	0.18	0.17	0.37	0.5	0.48		0.5	0.42	0.48	0.23	0.14	0.14	0.14	0.12
BE30	0.34	0.3	0.23	0.28	0.21	0.19	0.42	0.55		0.49	0.59	0.56	0.6	0.26	0.16	0.16	0.16	0.13
BE29	0.35	0.33	0.27	0.34	0.23	0.19	0.46		0.51	0.49	0.6	0.55	0.64	0.3	0.18	0.18	0.19	0.15
BE20	0.52	0.47	0.33	0.29	0.2	0.2		0.4	0.37	0.34	0.38	0.44	0.48	0.35	0.2	0.2	0.21	0.16
BE16	0.21	0.18	0.15	0.21	0.16		0.22	0.23	0.2	0.21	0.21	0.25	0.29	0.17	0.11	0.11	0.12	0.09
BE15	0.2	0.19	0.16	0.63		0.14	0.23	0.27	0.2	0.2	0.22	0.24	0.25	0.19	0.12	0.12	0.12	0.1
BE14	0.23	0.22	0.18		0.52	0.16	0.27	0.34	0.24	0.24	0.25	0.29	0.33	0.22	0.13	0.13	0.14	0.11
BE05	0.36	0.52		0.19	0.16	0.16	0.35	0.26	0.24	0.23	0.22	0.25	0.29	0.33	0.18	0.18	0.19	0.14
BE04	0.38		0.43	0.21	0.17	0.16	0.39	0.28	0.26	0.24	0.23	0.28	0.32	0.37	0.19	0.19	0.2	0.16
BE02		0.5	0.39	0.27	0.2	0.19	0.63	0.37	0.34	0.33	0.33	0.37	0.42	0.36	0.22	0.22	0.24	0.17

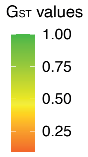

Figure S6: A) Relative migration network (G_{ST}) for *G. fossarum*. Only values > 0.3 are shown. All values are shown in the heatmap (B) with rows as source and columns sink populations.
